# Compact Micro-Coriolis Mass-Flow Meter with Optical Readout

**DOI:** 10.3390/mi15010114

**Published:** 2024-01-10

**Authors:** Mahdieh Yariesbouei, Remco G. P. Sanders, Remco J. Wiegerink, Joost C. Lötters

**Affiliations:** 1 Integrated Devices and Systems, University of Twente, 7522 NB Enschede, The Netherlands; mahdieh.yari@gmail.com (M.Y.); r.g.p.sanders@utwente.nl (R.G.P.S.); j.c.lotters@utwente.nl (J.C.L.); 2 Bronkhorst High-Tech BV, 7261 AK Ruurlo, The Netherlands

**Keywords:** compact micro-Coriolis mass-flow meter, nickel electroplated tube, optical readout

## Abstract

This paper presents the first nickel-plated micro-Coriolis mass-flow sensor with integrated optical readout. The sensor consists of a freely suspended tube made of electroplated nickel with a total length of 60 mm, an inner diameter of 580 µm, and a wall thickness of approximately 8 µm. The U-shaped tube is actuated by Lorentz forces. An optical readout consisting of two LEDs and two phototransistors is used to detect the tube motion. Mass-flow measurements were performed at room temperature with water and isopropyl alcohol for flows up to 200 g/h and 100 g/h, respectively. The measured resonance frequencies were 1.67 kHz and 738 Hz for water and 1.70 kHz and 752 Hz for isopropyl alcohol for the twist and swing modes, respectively. The measured phase shift between the two readout signals shows a linear response to mass flow with very similar sensitivities for water and isopropyl alcohol of $0.41\frac{\mathrm{mdeg}}{g/h}$ and $0.43\;\frac{\mathrm{mdeg}}{g/h}$, respectively.

## 1. Introduction

**Figure 1:**
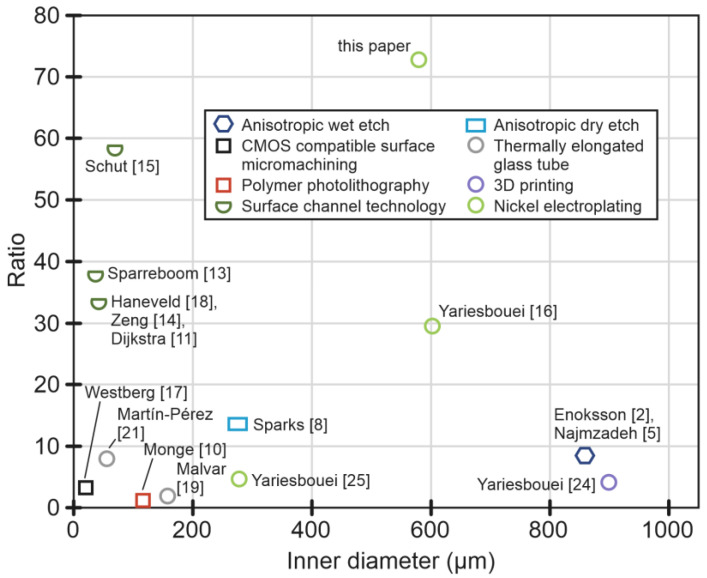
The ratio of the tube inner diameter to wall thickness versus the equivalent inner diameter of the cross-section for different fabrication methods. The marker used for each fabrication method corresponds to the resulting cross-sectional shape of the tube [2,5,8,10,11,13,14,15,16,17,18,19,21,24,25].

A Coriolis mass-flow and density sensor detects mass flow by utilizing the secondary vibration induced by the Coriolis forces resulting from fluid flow inside a vibrating tube. The amplitude of this secondary vibration is directly proportional to mass flow. The fluid density is obtained from the resonance frequency of the tube. Compared to other flow-sensing principles, Coriolis mass-flow sensors have the advantage of being independent of the type of fluid, but they are relatively large [1]. Twenty-five years ago, the first microfluidic density and micro-Coriolis mass-flow sensor was introduced [2]. Subsequently, a multitude of micro-Coriolis mass-flow sensors have been presented based on micromachining fabrication techniques, in which the tube cross-sectional shape was defined by, e.g., anisotropic wet [2,3,4,5] or dry [6,7,8,9] etching of silicon, polymer photolithography [10], and surface channel technology [11,12,13,14,15]. All these fabrication methods lead to a freely suspended channel with a non-circular cross-sectional shape, such as hexagonal, rectangular, or semicircular, and a limited range of wall thicknesses and cross-sectional areas. As will be shown in Section 2, to obtain a high sensitivity for fluid density and mass flow, the mass of the channel should be small compared to the mass of the fluid. Therefore, the inner diameter or cross-sectional area of the channel should be large compared to the wall thickness [16], or a low-density wall material should be chosen. A large channel diameter also has the advantage that it results in a low pressure drop over the sensor. Figure 1 shows an overview of the reported diameter-to-wall-thickness ratios as a function of the diameter for the different aforementioned methods. For non-circular channel cross-sections, an equivalent diameter is calculated, which corresponds to the same cross-sectional area. For a given diameter, a larger diameter-to-wall-thickness ratio will result in improved sensitivity due to the relatively reduced mass of the wall with respect to the mass of the fluid. However, other factors, like the readout method that is used to detect the channel motion, also affect the sensitivity, so a direct comparison of the sensitivities is not possible. The first silicon micromachined sensor proposed by Enoksson [2] gives a diameter-to-wall-thickness ratio of 9. Surface micromachined rectangular cross-section tubes, as proposed by Westberg [17], typically result in a relatively poor ratio due to the limited thickness of the sacrificial layer. Tubes with rectangular cross-sections were also proposed by Sparks [8], who achieved an equivalent diameter-to-wall-thickness ratio of about 14. Dijkstra [11] proposed a semicircular tube with a thin silicon-rich silicon nitride tube wall. The diameter of 40 μm and wall thickness of 1.2 μm result in a diameter-to-wall-thickness ratio of 33. However, in this case, the flat top side of the channel does result in some pressure dependence [18].

To prevent channel deformations due to pressure, particularly when the tube wall is relatively thin, it is necessary to have a circular cross-section for the channel [16]. In this regard, some devices were developed based on glass microcapillaries [19,20,21,22,23], 3D printing [24], and nickel electroplating [16]. The proposed glass microcapillaries are made by thermally elongating glass tubes. The inner and outer diameters are adjusted by changing factors like laser power or pulling force, but there is a limit to how thin the walls can be in relation to the inner diameter. In [21], a diameter-to-wall-thickness ratio of 8.5 was achieved. Three-dimensionally printed channels were reported in [24] with a ratio up to 3.9. In this case, the maximum ratio was limited by the smallest wall thickness of the tube that can be reliably printed without resulting in leakage. This thickness depends on different parameters, such as tube structure, dimensions, precision of the printer, and the resin. For nickel electroplated tubes [16,25], a ratio of 30 was demonstrated. This method allows the fabrication of tubes with a minimum wall thickness of 8 μm and a minimum diameter of 120 μm. In this paper, we use this method for fabricating a tube with a diameter of 580 µm and a wall thickness of 8 µm, resulting in a ratio of more than 70.

Initial mass-flow measurements on sensors based on nickel electroplated tubes have shown that the devices also work as Coriolis mass-flow sensors [16,25], although poor accuracy and repeatability were achieved. Furthermore, a laser Doppler vibrometer was used to detect the tube movement, but such an instrument cannot be integrated into a compact flow meter. Therefore, in this paper, we present the design and realization of a sensor with an integrated optical readout consisting of two LEDs and two phototransistors.

The paper is organized as follows. In Section 2, the operation principle of the micro-Coriolis mass-flow sensor and the analytical model of the sensor are explained. Section 3 describes the design, the fabrication method, and the completely assembled device. Measurement results and a comparison between the analytical model and the practical results are presented in Section 4.

## 2. Operation Principle

**Figure 2:**
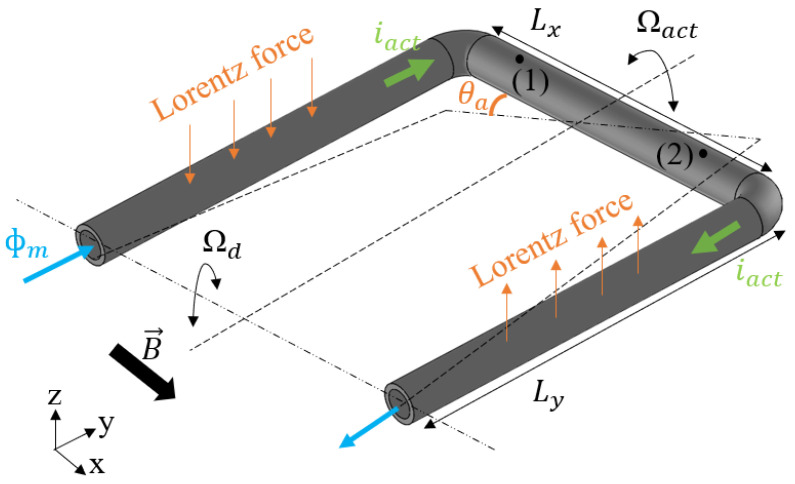
Basic operation principle of micro-Coriolis mass-flow sensing. A U-shaped tube is actuated in the twist mode around the y axis, indicated by angular velocity $\Omega_{act}$. A mass flow $\Phi_{m}$ inside the tube will result in Coriolis forces in the tube segment with length $L_{x}$, which actuate a swing mode vibration around the x axis, indicated by angular velocity $\Omega_{d}$. The tube displacement is measured at two points marked as (1) and (2) to detect the amplitudes of both the twist and swing mode vibrations.

Figure 2 shows the basic operation principle of micro-Coriolis mass-flow sensing. The Coriolis mass-flow sensor presented in this paper contains a freely suspended U-shaped tube that is brought into vibration by Lorentz force in a twist mode around the y axis, indicated by $\Omega_{act}$. An applied flow inside the vibrating tube will induce a second vibration in swing mode, indicated by $\Omega_{d}$ around the x axis due to the resulting Coriolis forces. The Coriolis forces are linearly dependent on the mass-flow rate, so the amplitude of the second vibration mode is a measure of the mass-flow. The tube displacement in the z direction is measured in two points, marked as (1) and (2) in Figure 2. The differential displacement in these points is a measure of the twist mode movement that is actuated by Lorentz force. The common displacement is a measure of the swing mode movement due to the Coriolis forces. As will be shown below, the phase difference between the motion in points (1) and (2) is a measure of the mass flow.

Based on the analytical model of a U-shaped Coriolis mass-flow sensor presented in [24], the mass-flow sensitivity can be expressed as:

(1) $$S=\frac{{\hat{z}}_{d}}{{\hat{\theta }}_{a}\Phi_{m}}=\frac{1}{m}\frac{2L_{x}\left(L_{x}+2L_{y}\right)}{L_{x}+\frac{2}{3}L_{y}}\frac{Q_{d}\omega_{a}}{\sqrt{{\left(\omega_{d}^{2}-\omega_{a}^{2}\right)}^{2}Q_{d}^{2}+\omega_{d}^{2}\omega_{a}^{2}}}\;,$$

where ${\hat{z}}_{d}$ is the amplitude of the displacement in z direction of the tube segment with length $L_{x}$ due to the Coriolis forces, ${\hat{\theta }}_{a}\;$is the amplitude of the angular displacement in the actuation mode, $\Phi_{m}$ is the mass-flow rate, and m is the total mass of the tube and the fluid inside the tube. $L_{x}$ and $L_{y}$ are the length of the tube in x and y direction as indicated in Figure 2, $\omega_{a}$ and $\omega_{d}$ are the resonance frequencies of the tube in actuation (twist) and detection (swing) mode, respectively, and $Q_{d}$ is the quality factor for the detection mode.

The sensor is typically actuated at the resonance frequency $\omega_{a}$ to obtain a large actuation amplitude. If the detection mode resonance frequency $\omega_{d}$ is sufficiently different from the actuation frequency, i.e., when

(2) $$\left(\omega_{d}^{2}-\omega_{a}^{2}\right)Q_{d}\gg \omega_{d}\omega_{a},$$

Equation (1) can be simplified to:

(3) $$S=\frac{{\hat{z}}_{d}}{{\hat{\theta }}_{a}\Phi_{m}}=\frac{1}{m}\frac{2L_{x}\left(L_{x}+2L_{y}\right)}{L_{x}+\frac{2}{3}L_{y}}\frac{\omega_{a}}{\left|\omega_{d}^{2}-\omega_{a}^{2}\right|}\;.$$

We see that the sensitivity decreases with the total mass m, showing the importance of having a thin tube wall or using a low-density tube material. The dimensions $L_{x}$ and $L_{y}$ of course, affect the resonance frequencies and, therefore, cannot be chosen independently from $\omega_{a}$ and $\omega_{d}$. In general, we see that higher resonance frequencies will result in lower sensitivity because the difference $\left|\omega_{d}^{2}-\omega_{a}^{2}\right|$ will also increase to fulfill Equation (2). Haneveld [12] showed that for a quality factor of $Q_{d}=30$, the ratio between the resonance frequencies should be at least 1.4 so that a change in quality factor does not significantly affect the sensitivity given by (3). If the ratio between the resonance frequencies is 1.4, a change in $Q_{d}$ from 30 to $10^{4}$ will only change the sensitivity by 0.1% [12].

**Figure 3:**
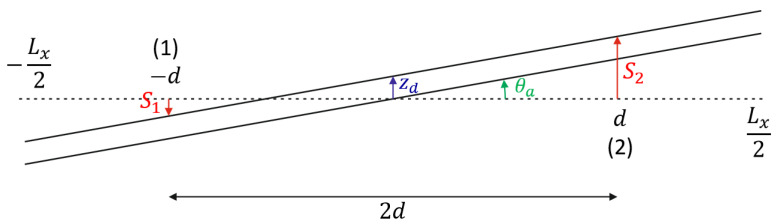
Schematic drawing showing the displacement of the tube segment with length $L_{x}$ due to a combination of an actuation angle $\theta_{a}$ and a displacement due to Coriolis forces $z_{d}$. The optical readout measures the displacements $S_{1}$ and $S_{2}$, at the points (1) and (2), which are at a distance d from the center.

Figure 3 shows a schematic drawing of the displacement of the tube segment with length $L_{x}$ due to a combination of an actuation angle $\theta_{a}$ and a displacement due to Coriolis forces $z_{d}$. The displacement of the tube is measured at points (1) and (2), which are at a distance d from the center. The actuation angle can be expressed as a function of time [14]:

(4) $$\theta_{a}(t)={\hat{\theta }}_{a}cos(\omega_{a}t+\theta_{0}),$$

with ${\hat{\theta }}_{a}$ the actuation amplitude, and $\theta_{0}$ an arbitrary phase shift. The Coriolis forces are proportional to the angular velocity, and the resulting displacement can, therefore, be expressed as:

(5) $$z_{d}(t)={\hat{z}}_{d}sin(\omega_{a}t+\theta_{0}),$$

with ${\hat{z}}_{d}$ the amplitude of the Coriolis displacement.

Based on Figure 3, we can now express the displacements $S_{1}(t)$ and $S_{2}(t)$ at the measurement points (1) and (2) as:

(6) $$S_{1}(t)=-d\cdot \theta_{a}(t)+z_{d}(t)=A\;sin(\omega_{a}t+{\theta +\theta }_{0}-\frac{\pi }{2}),$$

(7) $$S_{2}(t)=d\cdot \theta_{a}(t)+z_{d}(t)=A\;sin(\omega_{a}t-{\theta +\theta }_{0}+\frac{\pi }{2}),$$

with:

(8) $$A=\frac{1}{2}\sqrt{{\left(2d\;\tan{\hat{\theta }}_{a}\right)}^{2}+{\left(2{\hat{z}}_{d}\right)}^{2}},\;\mathrm{and}$$

(9) $$\theta =\mathrm{atan}\left(\frac{{\hat{z}}_{d}}{d\;\tan{\hat{\theta }}_{a}}\right).$$

or, because ${\hat{z}}_{d}\ll d\tan{\hat{\theta }}_{a}$ and ${\hat{\theta }}_{a}$ is small:

(10) $$\theta =\frac{{\hat{z}}_{d}}{d\;\tan{\hat{\theta }}_{a}}=\frac{{\hat{z}}_{d}}{d\;{\hat{\theta }}_{a}}.$$

Thus, we see that the phase shift θ between the measured displacements at points (1) and (2) is proportional to ${\hat{z}}_{d}$, and, therefore, proportional to the mass flow $\Phi_{m}$.

By substituting ${\hat{z}}_{d}/{\hat{\theta }}_{a}$ from Equation (3) into Equation (10), we find that the phase shift θ between the displacements $S_{1}(t)$ and $S_{2}(t)$ can be expressed as:

(11) $$\theta =\frac{1}{d}\frac{1}{m}(\frac{2L_{x}\left(L_{x}+2L_{y}\right)}{L_{x}+\frac{2}{3}L_{y}}\frac{\omega_{a}}{\left|\omega_{d}^{2}-\omega_{a}^{2}\right|})\Phi_{m}$$

## 3. Design, Fabrication and Assembly of a Demonstrator Device

As explained above, high sensitivity can be achieved using a tube with a large diameter-to-wall-thickness ratio. In this way, a significant part of the vibrating mass is formed by the mass of the fluid, which improves the sensor’s sensitivity. Therefore, we designed a demonstrator mass-flow sensor with a tube diameter of 580 μm and a wall thickness of 8 μm, resulting in a diameter-to-wall-thickness ratio of 72.5. Compared to the mass-flow sensors presented in [16,25], the ratio is much higher, see Figure 1. Furthermore, due to the relatively large diameter, which is similar to [16], this demonstrator will have a high flow range and low pressure drop. Compared to [16], the dimensions $L_{x}$ and $L_{y}$ increased from $L_{x}=L_{y}=12$ mm to $L_{x}=L_{y}=20$ mm. The longer tube will result in a higher sensitivity and lower resonance frequencies $\omega_{a}$ and $\omega_{d}$, at the expense of a larger pressure drop. Furthermore, the longer length $L_{x}$ provides more space to integrate the optical readout.

**Figure 4:**
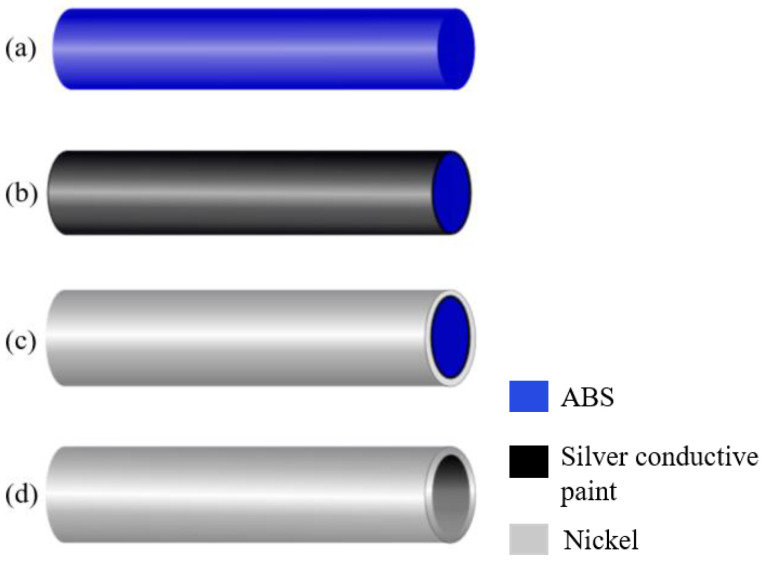
Schematic representation of the electroplating method. (**a**) ABS wire, (**b**) apply a coating of silver conductive paint, (**c**) nickel electroplating on the surface, (**d**) etch ABS wire. Reprinted with permission from Ref. [25]. ©2022, IEEE.

**Figure 5:**
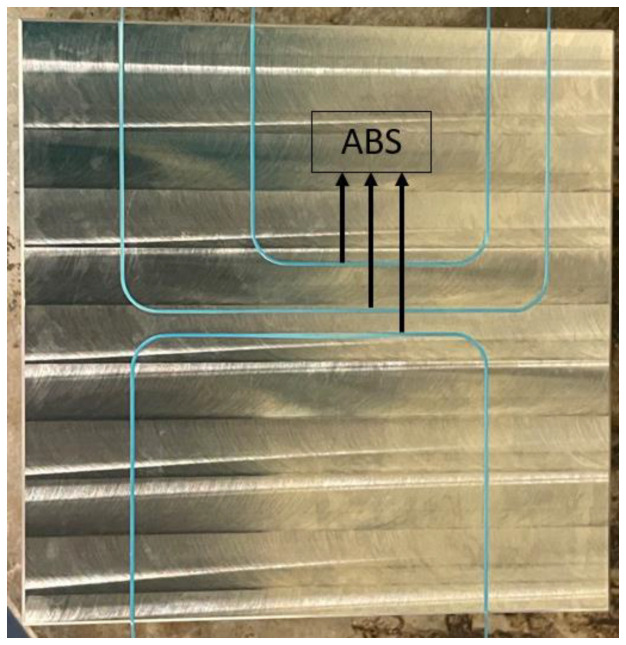
Stainless-steel mold containing grooves to bend the ABS. To bend the ABS without breaking the wire, the mold should be warmed up on a hotplate at around 80 °C before pressing the ABS wire into the groove.

**Figure 6:**
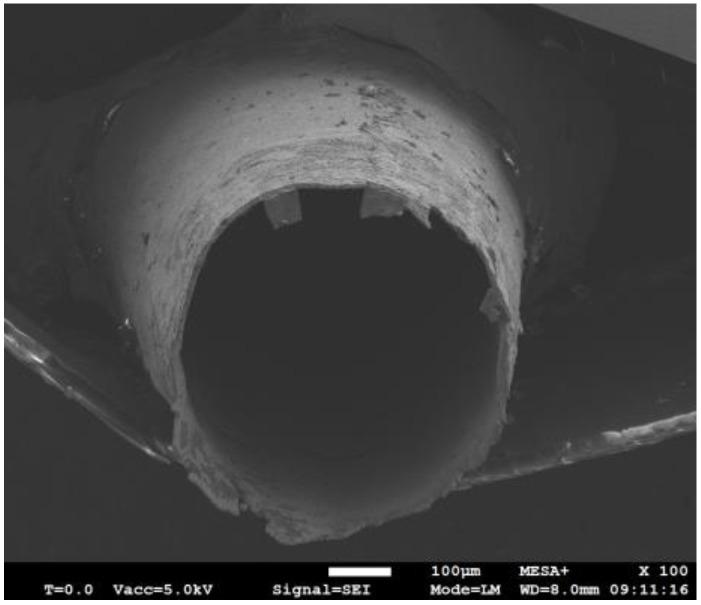
SEM photograph of the cross-section of the sensor tube with a diameter of 580 µm and wall of approximately 8 µm thick electroplated nickel.

The fabrication process of the tube is based on nickel electroplating of a pre-shaped acrylonitrile butadiene styrene (ABS) wire, as described in [16]. A summary of the process is given in Figure 4. In [16], we presented a mold to pre-shape the ABS wire. This mold could cause asymmetries in the resulting shape. Therefore, a new mold was designed, which is shown in Figure 5. This mold contains grooves with a semicircular cross-section with the same radius as the ABS wire. The grooves are made by milling on a stainless-steel block. To bend the ABS wire without breaking, first, the mold should be warmed up on a hotplate at around 80 °C. Next, the ABS wire is pressed inside the groove. Then, the shaped ABS wire, Figure 4a, is dipped in diluted silver conductive paint and dried at room temperature, Figure 4b. In the next step, the conductive ABS wire is coated with nickel in an electroplating bath, Figure 4c. Finally, the nickel-coated ABS wire is immersed in a beaker of acetone to dissolve the ABS wire, Figure 4d. Figure 6 shows an SEM picture of the cross-section of the nickel-plated tube.

**Figure 7:**
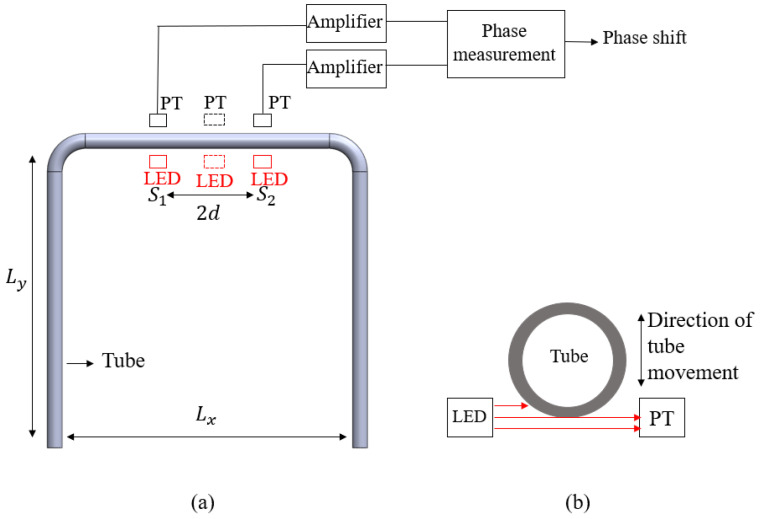
The configuration of the LEDs and phototransistors (PTs): (**a**) top view, (**b**) side view. The tube movement modulates the amount of received light. The phototransistor currents are amplified and converted into a voltage. In the current system, only the outer two LEDs and PTs are used. The phase difference between the two signals is a measure of the mass flow as given by Equation (11).

After preparing the free-suspended tube, the tube is soldered in a printed circuit board (PCB) and clamped onto a base containing stainless-steel tubes for fluidic connections. Figure 7 shows the design of the optical readout. Two or three LEDs are positioned at the inside of the U-shaped tube, as shown in Figure 7a. Two or three phototransistors (PTs) at the outside receive the light, which is modulated by the tube movement as indicated in Figure 7b. In the current system, only the outer two LED/PT pairs are actually used. The middle pair is optional and can be used for more accurate detection of tube movement.

**Figure 8:**
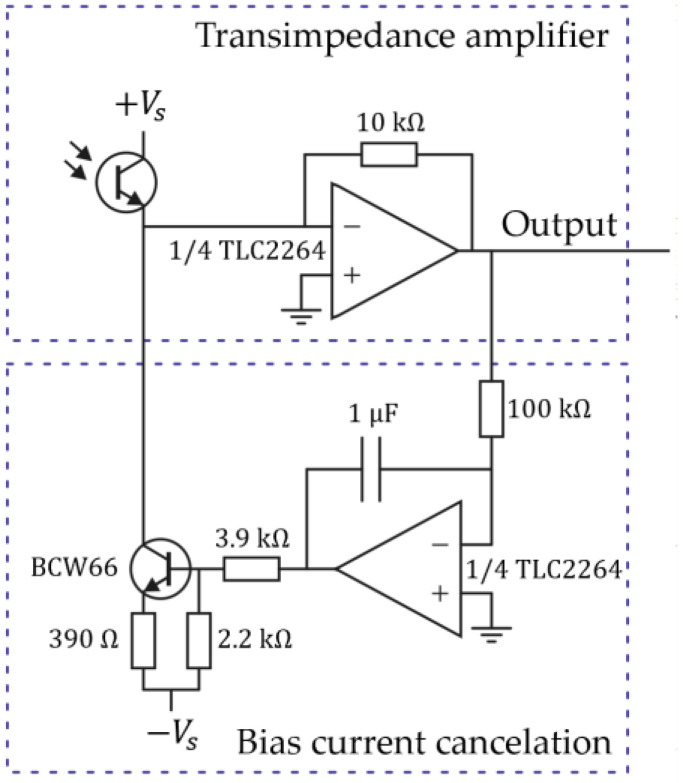
Schematic of the electronic circuit used to amplify the phototransistor current. The photocurrent resulting from light that is not modulated by the tube movement is subtracted using the bias current cancelation loop.

The LEDs are right-angle LEDIRs, type KPA3010F3C, with a peak spectral wavelength of 940 nm. WL-STSW SMT side-view phototransistors are used with a peak sensitivity at the same wavelength. The distance between the LEDs is 10 mm, i.e., d=5 mm. The phototransistor currents are amplified using the circuit shown in Figure 8. The trans-impedance amplifier converts the input current into a voltage using the feedback resistance of 10 kΩ. A bias current cancelation loop is added to keep the average output voltage at ground potential by subtracting the low-frequency part from the phototransistor current. The phase shift between the two amplified phototransistor signals is directly proportional to the mass flow in the tube, as given by (11). To measure the phase shift, we use a TiePie HS5 two-channel USB oscilloscope (TiePie engineering, Sneek, The Netherlands). The oscilloscope samples the signals with a sampling rate of 625 kSa/s and 16-bit resolution over a period of 300 ms, corresponding to approximately 500 signal periods, and the phase shift is calculated from the sampled signals.

**Figure 9:**
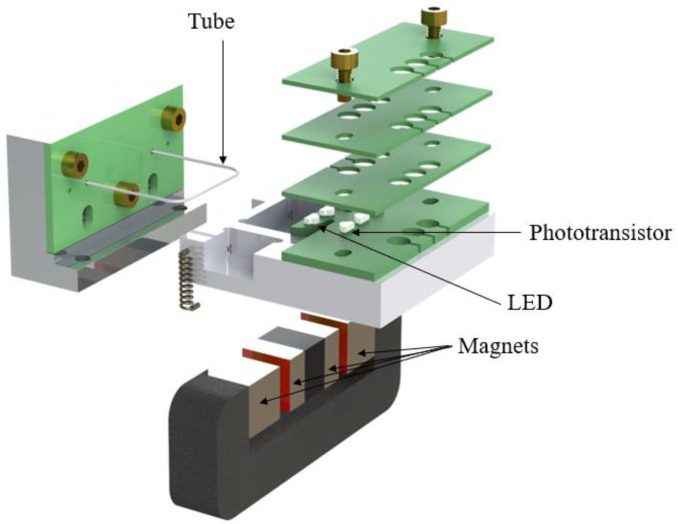
Exploded view of the demonstrator device. The tube is soldered in a PCB, which is clamped to an aluminum base containing stainless-steel tubes for fluidic connections. The LEDs and phototransistors are on a second PCB that is attached to the base through another aluminum piece that also contains a magnetic yoke with magnets to provide the magnetic field for actuation.

**Figure 10:**
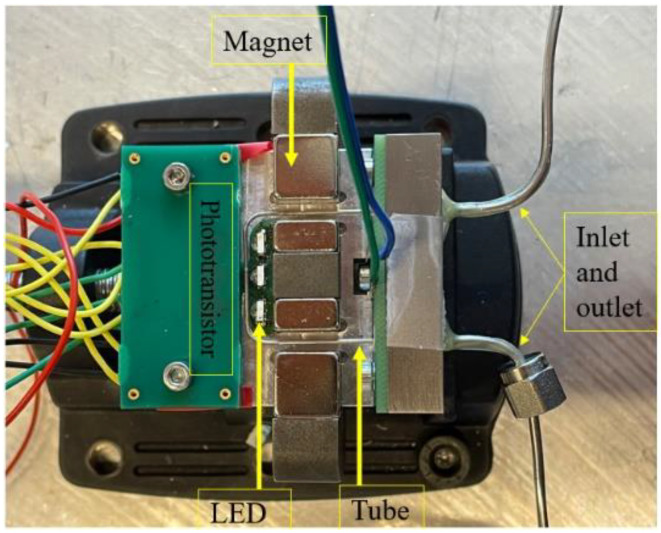
Photograph of the completely assembled mass-flow sensor.

Figure 9 shows an exploded view of the complete demonstrator device. As mentioned above, the U-shaped nickel tube is soldered on a PCB and clamped onto an aluminum base plate containing steel tubing for fluidic connection. A second aluminum part contains permanent magnets and a magnetic yoke to provide the magnetic field for Lorentz force actuation, as well as a PCB containing the phototransistors and LEDs. Three additional PCBs are stacked on top of the PCB containing the phototransistors, such that only the light from the corresponding LED can reach the transistor. Figure 10 shows a photograph of the completely assembled device.

## 4. Measurement Results and Discussion

**Figure 11:**
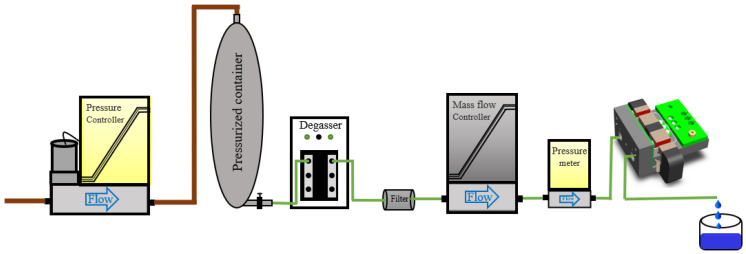
Block schematic of the fluidic measurement setup.

The measurement setup is shown in Figure 11. In this setup, the pressure controller sets the input pressure for the flow. The degasser and filter are used to eliminate bubbles and particles from the fluids. A mini-CORI-FLOW ML120 mass flow controller from Bronkhorst High-Tech BV, Ruurlo, The Netherlands, with an accuracy of ±0.2% is used to apply a well-defined mass-flow. An EL-PRESS pressure meter from Bronkhorst High-Tech BV is used to measure the pressure drop over the sensor during the measurement.

**Figure 12:**
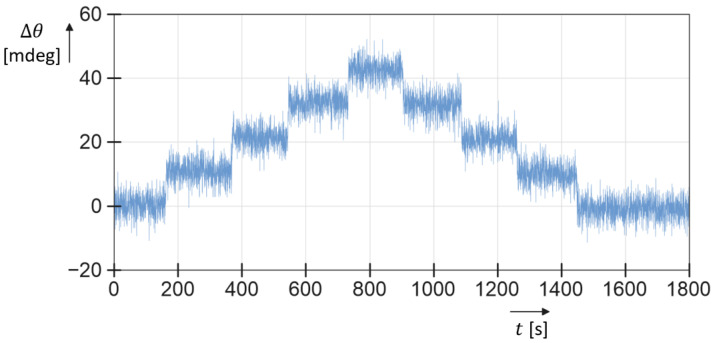
Typical measurement result showing the measured phase difference as a function of time while mass flow of IPA is changed from 0 to 100 g/h and back to 0 in steps of 25 g/h. The sampling time is 300 ms.

Measurements were performed at room temperature with a mass flow of water (density 998 kg/m^3^), 0–200 g/h, and isopropyl alcohol (density 793 kg/m^3^), 0–100 g/h, resulting in measured resonance frequencies of 1668.4 Hz and 738 Hz, and 1701.5 Hz and 752 Hz for the twist and swing mode, respectively. The input pressure on the pressurized container was set to 5 bar. The mass flow was applied using the reference Coriolis mass-flow controller, as shown in Figure 11. It is important to note that the measured flow range in this research was limited to 200 g/h for water and 100 g/h for IPA due to the maximum flow rate of the available mass-flow controller in the experimental setup. During the measurements, the mass flow was adjusted in steps of 20 or 25 g/h up to the maximum flow rate. After each step, the mass flow was kept constant for 2 to 3 min. Figure 12 shows the measured phase difference between the output signals of the outer two phototransistors with the mass flow of IPA as a function of time. We see that the sensor responds quickly to a change in mass flow, but there is significant noise in the phase measurement. Most likely, this noise is due to the large amount of light that can pass underneath the sensor tube and the relatively small modulation of the light due to the tube movement. The bias current cancelation (see Figure 8) removes the DC, but it will not remove the associated noise. To reduce the noise, the amount of light passing underneath the tube that is not modulated should be reduced. The signal-to-noise ratio can also be improved by reducing the distance d between the LED/PT pairs (see Figure 7). Removing the middle LED/PT pair would reduce d and increase the measured phase shift by a factor of 2.

**Figure 13:**
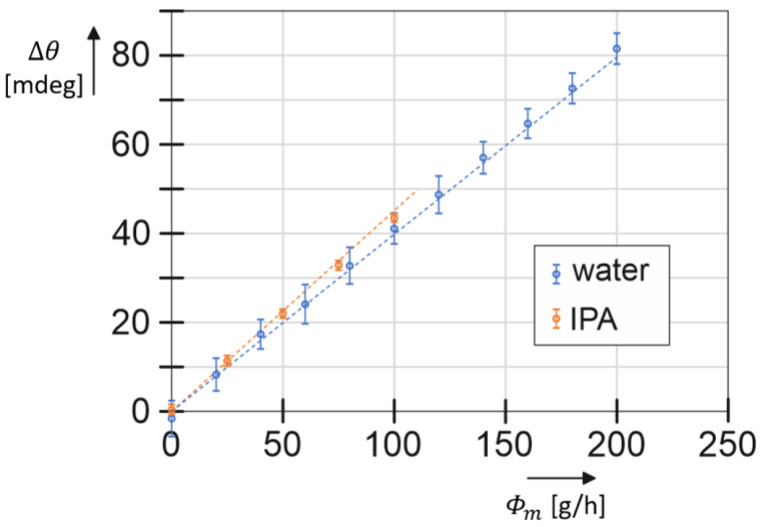
The output of the optical readout versus mass-flow rate. Noise in the measurement data was reduced by taking a moving average over 10 samples. The plot shows the mean of the measured phase difference at each flow rate. The error bars correspond to the standard deviation. The dashed lines show the theoretical response based on Equation (11).

Figure 13 shows the measured phase difference as a function of the applied mass flow for both water and IPA. The noise in the raw measurement data was reduced by taking a moving average over 10 samples, increasing the sampling time to 3 s. The figure shows the mean value of the phase difference at each measured flow rate. The error bars indicate the standard deviation in the measured values. A linear response is observed with very similar sensitivities for water and IPA of $4.1\cdot 10^{-4}\;\frac{\mathrm{degree}}{g/h}$ and $4.3\cdot 10^{-4}$ $\frac{\mathrm{degree}}{g/h}$, respectively. A small difference in slope can be expected because of the term $\frac{\omega_{a}}{\left|\omega_{d}^{2}-\omega_{a}^{2}\right|}$ in Equation (11), which causes the sensitivity to be dependent on the resonance frequencies and, therefore, on the fluid density. The dashed lines in Figure 13 show the predicted response from (11) with the measured resonance frequencies. For IPA, the sensitivity seems slightly lower, and for water, it is slightly higher than predicted. The exact reason for this small deviation needs to be further investigated.

At the maximum mass-flow rate, the measured pressure drop over the sensor is about 0.2 bar, which is the combined pressure drop of the sensor and the connecting tubing. This change in pressure had a negligible effect on the resonance frequencies. Thanks to the circular tube cross-section, the resonance frequencies changed by less than 0.1 Hz over the entire flow range.

## 5. Conclusions

In this paper, the first complete Coriolis mass-flow sensor is presented that is based on an electroplated nickel tube and contains an integrated optical readout. The free-standing nickel tube is fabricated by the electroplating method with a tube diameter of 580 μm and a wall thickness of 8 μm, giving a diameter-to-wall thickness ratio of 73:1. The tube has a total length of 60 mm. Thanks to the large diameter, the maximum pressure drop in the measured flow range of 0–100 g/h for IPA and 0–200 g/h for water is approximately 0.2 bar for the sensor, including connecting tubing.

The sensor is actuated by Lorentz force, using permanent magnets to provide the magnetic field and an actuation current through the tube wall. The integrated optical readout consists of two light emitters and two detectors. A twist mode vibration results in a differential modulation, and a swing mode vibration results in a common modulation of the light intensity. The phase difference between the detected signals is linearly dependent on the applied mass flow, with very similar sensitivity for water and IPA. The sensitivity for water is equal to $4.1\cdot 10^{-4}\;\frac{\mathrm{degree}}{g/h}$ and the sensitivity for IPA is equal to $4.3\cdot 10^{-4}$ $\frac{\mathrm{degree}}{g/h}$.

The presented sensor is much smaller than current commercially available devices. However, significant improvement will be needed to reach the same accuracy of 0.2%. In this research, the motion of the tube was detected using only two measurement points. As a potential future improvement, it is advisable to explore the detection of tube motion at three points or move the two measurement points closer together to increase the signal-to-noise ratio in the phase measurement. Furthermore, the amount of light passing underneath the tube that is not modulated by movement should be reduced to reduce the noise. Additionally, it is worth investigating alternative methods to dissolve ABS inside the tube because this step is time-consuming and can potentially damage the tube when left in an ultrasound bath for an extended period of time.

## Data Availability

Data are contained within the article.

## References

[1] Wang T., Baker R. (2014). Coriolis flowmeters: A review of developments over the past 20 years, and an assessment of the state of the art and likely future directions. Flow Meas. Instrum..

[2] Enoksson P., Stemme G., Stemme E. (1995). Fluid density sensor based on resonance vibration. Sens. Actuators A Phys..

[3] Enoksson P., Stemme G., Stemme E. A Coriolis mass flow sensor structure in silicon. Proceedings of the Ninth International Workshop on Micro Electromechanical Systems.

[4] Enoksson P., Stemme G., Stemme E. (1997). A silicon resonant sensor structure for Coriolis mass-flow measurements. J. Microelectro-Mech. Syst..

[5] Najmzadeh M., Haasl S., Enoksson P. (2007). A silicon straight tube fluid density sensor. J. Micromech. Microeng..

[6] Zhang Y., Tadigadapa S., Najafi N. (2001). A micromachined Coriolis-force-based mass flowmeter for direct mass flow and fluid density measurement. Transducers’01 Eurosensors XV.

[7] Sparks D., Smith R., Massoud-Ansari S., Najafi N. Coriolis mass flow, density and temperature sensing with a single vacuum sealed MEMS chip. Proceedings of the Solid-State Sensor, Actuator and Microsystems Workshop.

[8] Sparks D., Smith R., Straayer M., Cripe J., Schneider R., Chimbayo A., Anasari S., Najafi N. (2003). Measurement of density and chemical concentration using a microfluidic chip. Lab Chip.

[9] Smith R., Sparks D.R., Riley D., Najafi N. (2008). A MEMS-based Coriolis mass flow sensor for industrial applications. IEEE Trans. Ind. Electron..

[10] Monge R., Groenesteijn J., Alveringh D., Wiegerink R.J., Lötters J., Fernandez L.J. (2017). SU–8 micro Coriolis mass flow sensor. Sens. Actuators B Chem..

[11] Dijkstra M., de Boer M.J., Berenschot J.W., Lammerink T.S., Wiegerink R.J., Elwenspoek M. (2007). A versatile surface channel concept for microfluidic applications. J. Micromech. Microeng..

[12] Haneveld J., Lammerink T.S., de Boer M.J., Sanders R.G., Mehendale A., Lötters J.C., Dijkstra M., Wiegerink R.J. (2010). Modeling, design, fabrication and characterization of a micro Coriolis mass flow sensor. J. Micromech. Microeng..

[13] Sparreboom W., Van de Geest J., Katerberg M., Postma F., Haneveld J., Groenesteijn J., Lammerink T., Wiegerink R., Lötters J. (2013). Compact mass flow meter based on a micro Coriolis flow sensor. Micromachines.

[14] Zeng Y., Groenesteijn J., Alveringh D., Wiegerink R.J., Lötters J.C. (2021). Design, fabrication, and characterization of a micro Coriolis mass flow sensor driven by PZT thin film actuators. J. Microelectromech. Syst..

[15] Schut T., Wiegerink R., Lötters J. (2020). μ-Coriolis mass flow sensor with resistive readout. Micromachines.

[16] Yariesbouei M., Sanders R.G., Moazzenzade T., Wiegerink R.J., Lötters J.C. (2022). Free suspended thin-walled nickel electroplated tubes for microfluidic density and mass flow sensors. J. Microelectromech. Syst..

[17] Westberg D., Paul O., Andersson G.I., Baltes H. A CMOS-compatible device for fluid density measurements. Proceedings of the IEEE the Tenth Annual International Workshop on Micro Electro Mechanical Systems. An Investigation of Micro Structures, Sensors, Actuators, Machines and Robots.

[18] Haneveld J., Lammerink T.S.J., Dijkstra M., Droogendijk H., de Boer M.J., Wiegerink R.J. Highly sensitive micro Coriolis mass flow sensor. Proceedings of the 2008 IEEE 21st International Conference on Micro Electro Mechanical Systems.

[19] Malvar O., Ramos D., Martínez C., Kosaka P., Tamayo J., Calleja M. (2015). Highly sensitive measurement of liquid density in air using suspended microcapillary resonators. Sensors.

[20] Lee D., Kim J., Cho N.J., Kang T., Kauh S., Lee J. (2016). Pulled microcapillary tube resonators with electrical readout for mass sensing applications. Sci. Rep..

[21] Martín-Pérez A., Ramos D., Gil-Santos E., García-López S., Yubero M.L., Kosaka P.M., San Paulo Á., Tamayo J., Calleja M. (2019). Mechano-optical analysis of single cells with transparent microcapillary resonators. ACS Sens..

[22] Martín-Pérez A., Ramos D., Tamayo J., Calleja M. (2021). Nanomechanical molecular mass sensing using suspended microchannel resonators. Sensors.

[23] Martín-Pérez A., Ramos D., Tamayo J., Calleja M. (2019). Coherent optical transduction of suspended microcapillary resonators for multi-parameter sensing applications. Sensors.

[24] Yariesbouei M., Sanders R.G., Wiegerink R.J., Lötters J.C. (2023). Modeling, Fabrication, and Testing of a 3D-Printed Coriolis Mass Flow Sensor. Sensors.

[25] Yariesbouei M., Sanders RG P., Wiegerink R.J., Lötters J.C. Micro Coriolis Mass Flow Sensor Based on Electroplated Nickel Tubes. Proceedings of the 2022 IEEE Sensors.

